# Reply to the letter of Soliman et al. regarding ‘Randomised comparison of a balloon-expandable and self-expandable valve with quantitative assessment of aortic regurgitation using magnetic resonance imaging’

**DOI:** 10.1007/s12471-020-01488-w

**Published:** 2020-09-08

**Authors:** N. H. M. Kooistra, M. Voskuil, P. R. Stella

**Affiliations:** Department of Cardiology, University Medical Centre Utrecht, Utrecht, The Netherlands

First of all we would like to thank Soliman et al. for their extensive reaction [1]. However, we believe there has been a misunderstanding concerning the purpose of the ELECT study results.

1. As mentioned in the Background section of our article [2], the use of quantitative phase-contrast magnetic resonance imaging (MRI) as a tool to accurately assess the severity of aortic valve regurgitation (AR) was tested in a clinical setting. Although quantitative aortography with videodensitometric assessment is indeed also a promising tool to accurately assess AR, an important limitation of this technique compared to MRI is that quantitative aortography is less suitable for (non-invasive) follow-up. We also do not encourage the use of MRI for a day-to-day routine assessment after transcatheter aortic valve implantation (TAVI). However, we suggest that it could be used as a research tool in the assessment of AR in future TAVI studies. The use of MRI as a research tool could further improve the quality of valve comparison studies, since the tool used in daily practice for the assessment of AR, i.e. echocardiography, often results in a wide range of under- or over-estimation and has a huge range of inter-observer variability.
  As stated in the Conclusion section: ‘Although the use of MRI to quantify AR is not feasible in daily clinical practice, it should be considered as a surrogate endpoint for clinical outcomes in comparative valve studies’.
2. Indeed, one of the drawbacks of the study was the fact that pacemaker implantation after TAVI prevented the use of MRI in 7 patients. As described, the other 6 patients were excluded from MRI analysis due to pre-procedural implantation of a pacemaker, MRI flow measuring error, mortality or clinical instability.

Of the 13 patients that were excluded from MRI analyses, we did assess the severity of AR in 11 patients by performing a transthoracic echocardiogram (TTE). No TTE was performed in 2 patients of the self-expandable valve (SEV) group due to procedural mortality. The following AR rates were collected by TTE at discharge. In the balloon expandable valve (BEV) group (*n* = 6), 33% of patients had mild AR, and 67% had trace AR or none. In the SEV group (*n* = 5), 40% of patients had moderate AR, and 60% had trace AR or none. These AR results of the excluded patients assessed with TTE do not contradict the reported results of the MRI analysis. Therefore, the potential effect of selection bias due to exclusion of these patients from MRI examination on the reported results appears to be limited.

3. The authors are right in their comment that atrial fibrillation reduces the accuracy of flow measurements on MRI. This is one of the limitations of MRI. The reported number of patients with atrial fibrillation at baseline included those with paroxysmal or chronic atrial fibrillation. The heart rhythm on ECG at discharge, which was measured close to the MRI examination, showed atrial fibrillation in 8 patients (5 BEV and 3 SEV). Indeed, the accuracy in these patients might have been reduced due to the atrial fibrillation.
4. Finally, the comment on first-generation SEVs versus later-generation BEVs was mentioned in our Limitations section. To clarify: ‘at the time of study design and enrolment these valves were the only commercially available and most frequently used SEV/BEV’. The next-generation SEV was introduced more than 2 years after the start of study enrolment and at that time—mainly due to slow patient recruitment (mostly refusal to participate in the study)—the decision was made to stop enrolment rather than start a second-phase trial. However, it has also been shown that the use of the second-generation CoreValve Evolut is still complicated by a relatively high number of pacemaker implantations [3], which may negate the potential positive clinical effect of reduced AR with the newer design of this valve.

In general, owing to the nature of a device study design, where a comparison is made in daily use, the results will always be debated a couple of years later when new devices with an improved design have been made available. However, we strongly believe that making these differences clear it will stimulate researchers and companies to improve and further develop their products to achieve the highest standards possible at that particular point in time.
